# British isles lupus assessment group 2004 index is valid for assessment of disease activity in systemic lupus erythematosus

**DOI:** 10.1002/art.23130

**Published:** 2007-12

**Authors:** Chee-Seng Yee, Vernon Farewell, David A Isenberg, Anisur Rahman, Lee-Suan Teh, Bridget Griffiths, Ian N Bruce, Yasmeen Ahmad, Athiveeraramapandian Prabu, Mohammed Akil, Neil McHugh, David D'Cruz, Munther A Khamashta, Peter Maddison, Caroline Gordon

**Affiliations:** 1 University of BirminghamBirmingham, UK; 2 MRC Biostatistics UnitCambridge, UK; 3 University College LondonLondon, UK; 4 Royal Blackburn HospitalBlackburn, UK; 5 Freeman HospitalNewcastle-upon-Tyne, UK; 6 University of ManchesterManchester, UK; 7 Manchester Royal InfirmaryManchester, UK; 8 Sheffield Teaching Hospitals NHS TrustSheffield, UK; 9 Royal National Hospital for Rheumatic Diseases NHS TrustBath, UK; 10 St Thomas' HospitalLondon, UK; 11 University of WalesBangor, UK

## Abstract

**Objective:**

To determine the construct and criterion validity of the British Isles Lupus Assessment Group 2004 (BILAG-2004) index for assessing disease activity in systemic lupus erythematosus (SLE).

**Methods:**

Patients with SLE were recruited into a multicenter cross-sectional study. Data on SLE disease activity (scores on the BILAG-2004 index, Classic BILAG index, and Systemic Lupus Erythematosus Disease Activity Index 2000 [SLEDAI-2K]), investigations, and therapy were collected. Overall BILAG-2004 and overall Classic BILAG scores were determined by the highest score achieved in any of the individual systems in the respective index. Erythrocyte sedimentation rates (ESRs), C3 levels, C4 levels, anti–double-stranded DNA (anti-dsDNA) levels, and SLEDAI-2K scores were used in the analysis of construct validity, and increase in therapy was used as the criterion for active disease in the analysis of criterion validity. Statistical analyses were performed using ordinal logistic regression for construct validity and logistic regression for criterion validity. Sensitivity, specificity, positive predictive value (PPV), and negative predictive value (NPV) were calculated.

**Results:**

Of the 369 patients with SLE, 92.7% were women, 59.9% were white, 18.4% were Afro-Caribbean and 18.4% were South Asian. Their mean ± SD age was 41.6 ± 13.2 years and mean disease duration was 8.8 ± 7.7 years. More than 1 assessment was obtained on 88.6% of the patients, and a total of 1,510 assessments were obtained. Increasing overall scores on the BILAG-2004 index were associated with increasing ESRs, decreasing C3 levels, decreasing C4 levels, elevated anti-dsDNA levels, and increasing SLEDAI-2K scores (all *P* < 0.01). Increase in therapy was observed more frequently in patients with overall BILAG-2004 scores reflecting higher disease activity. Scores indicating active disease (overall BILAG-2004 scores of A and B) were significantly associated with increase in therapy (odds ratio [OR] 19.3, *P* < 0.01). The BILAG-2004 and Classic BILAG indices had comparable sensitivity, specificity, PPV, and NPV.

**Conclusion:**

These findings show that the BILAG-2004 index has construct and criterion validity.

Assessment of disease activity in systemic lupus erythematosus (SLE) is challenging in view of the ability of SLE to affect any organ or system, resulting in diverse clinical manifestations. This is compounded by the lack of a biomarker that uniformly reflects disease activity well. As a result, numerous composite clinical indices have been developed for standardized assessment of disease activity (1).

The British Isles Lupus Assessment Group 2004 (BILAG-2004) index (2) was developed recently for the assessment of disease activity in SLE, and it represents a major revision of the Classic BILAG index (3). Like the Classic BILAG index, it is a transitional index that is able to capture changing severity of clinical manifestations. It is an ordinal scale index, which does not include a global score but instead produces an overview of disease activity across 9 systems. The interrater reliability of this index has been established and described elsewhere (2,4). The aim of this study was to determine the construct and criterion validity of the BILAG-2004 index in assessment of SLE disease activity.

## PATIENTS AND METHODS

### Study design

This was a multicenter cross-sectional study involving 8 centers in the UK. All patients included in the study were diagnosed as having SLE according to the American College of Rheumatology criteria (5,6). Patients were excluded from the study if they were pregnant, <18 years of age, or unable to give valid consent. This study was carried out in accordance with the Helsinki Declaration and received multicenter research approval from the Hull and East Riding Research Ethics Committee (Hull, UK) as well as approval from the local research ethics committees of all participating centers. Written consent was obtained from all patients.

The study was conducted from March 2005 to August 2006. At every assessment, data on disease activity, investigations, and treatment were collected. Disease activity was assessed using the BILAG-2004 index, Classic BILAG index, and Systemic Lupus Erythematosus Disease Activity Index 2000 (SLEDAI-2K) (7). All clinicians involved in this study had been trained to use all 3 disease activity indices. More than 1 assessment was obtained on the majority of patients during the study period.

### Classic BILAG index

The BILAG index is an ordinal scale index that assesses 8 systems (general, mucocutaneous, neuropsychiatric, musculoskeletal, cardiorespiratory, vasculitis, renal, and hematologic) (3). It was developed based on the principle of physician's intention to treat. Disease activity is categorized into 5 different levels from A to E. Grade A represents very active disease requiring immunosuppressive drugs and/or >20 mg of prednisolone or equivalent daily. Grade B represents moderately active disease requiring lower doses of glucocorticoids, antimalarials, or nonsteroidal antiinflammatory drugs (NSAIDs). Grade C indicates mild stable disease, while grade D indicates that there is no current disease activity but that the system had previously been affected. Grade E indicates no current or previous disease activity.

### BILAG-2004 index

Like the Classic BILAG index, this is an ordinal scale index based on the principle of physician's intention to treat. However, all of the items were revised and reclassified into 9 systems (constitutional, mucocutaneous, neuropsychiatric, musculoskeletal, cardiorespiratory, gastrointestinal, ophthalmic, renal, and hematologic). Disease activity is scored from A to E, similar to the Classic BILAG index. However, the scoring scheme was refined to reflect the fact that anticoagulation (in combination with intensive immunosuppression), topical glucocorticoids or immunosuppressive agents, thalidomide, prasterone, and retinoids may be used to treat active manifestations. Therefore, grade A also includes very active disease requiring anticoagulation therapy (in the presence of immunosuppressive drugs or high-dose steroids), and grade B also includes moderately active disease requiring topical steroids, topical immunosuppressive agents, thalidomide, prasterone, or retinoids.

### SLEDAI-2K

The SLEDAI-2K consists of 24 items, of which 16 are clinical and 8 are based solely on laboratory results (urinary casts, hematuria, proteinuria, pyuria, low complement levels, increased DNA binding, thrombocytopenia, and leukopenia) (7). A manifestation is recorded if it has been present at any point during the past 10 days, regardless of severity or whether it has improved or worsened. Weighting is used, resulting in individual item scores ranging from 1 to 8 and a global score ranging from 0 to 105. In the present study, items for which laboratory results were not available were scored as negative or normal.

### Statistical analysis

All statistical analyses were performed using Stata for Windows, version 8 (StataCorp, College Station, TX). For the purpose of the analysis, overall BILAG-2004 and overall Classic BILAG scores were used. These overall scores were determined by the highest score achieved in any system in the respective index. BILAG-2004 and Classic BILAG scores of D and E were combined, since both indicate inactivity. Therefore, 4 categorical overall scores were possible (A, B, C, and D).

#### Construct validity

The constructs used in this validation study were the erythrocyte sedimentation rate (ESR), C3 and C4 complement levels, anti–double-stranded DNA antibody (anti-dsDNA) level, and SLEDAI-2K score. It was hypothesized that the overall score on the BILAG-2004 index would have a positive correlation or association with the ESR, anti-dsDNA level, and SLEDAI-2K score (since they increase with disease activity), and a negative correlation or association with complement C3 and C4 levels (since they decrease with disease activity). ESR and levels of anti-dsDNA, C3, and C4 were determined locally at the participating centers. Since the laboratory kits used were not the same in all centers, the normal values for anti-dsDNA, C3, and C4 levels differed among centers. Therefore, for the purpose of analysis, these constructs were divided into ordinal categories. For ESR, the categories were normal (0–30 mm/hour), elevated (31–60 mm/hour), and markedly elevated (>60 mm/hour). For C3 and C4 levels, the categories were normal, low, and very low (less than or equal to half the lower limit of normal). For anti-dsDNA level, the categories were normal, elevated, and very high (>5 times the upper limit of normal), and for SLEDAI-2K score the categories were inactive (score of 0), mildly active (scores of 1–3), active (scores of 4–12), and very active (scores >12). The definitions of low C3 and C4 levels and elevated dsDNA level varied depending on the study center. Repeat analysis was performed using ESR and SLEDAI-2K scores as continuous variables.

Maximum-likelihood ordinal logistic regression was used to assess construct validity, with overall BILAG-2004 score as the outcome variable and the constructs as the explanatory variable. The normal or inactive category for each construct was used as a baseline comparator for the other categories. Since the majority of patients were assessed more than once, independence of observations from the same patient could not be assumed. Therefore, robust variance estimation (Huber/White/sandwich variance estimator) was used instead of the standard maximum-likelihood variance estimation (8). Results were reported as odds ratio (ORs) with 95% confidence intervals (95% CIs).

#### Criterion validity

Since there is no absolute gold standard for disease activity in SLE, change in therapy was used as the criterion. Change in therapy was defined as the difference in therapy after the patient was assessed compared with the therapy that the patient was receiving prior to assessment. Three categories of change were defined, namely, no change, increase in therapy, and decrease in therapy. Treatments of interest were immunosuppressive agents, antimalarials, glucocorticoids, biologic response modifiers, topical glucocorticoids, topical immunosuppressive agents, intravenous immunoglobulins, plasmapheresis, anticoagulation, prasterone, thalidomide, and retinoids. NSAIDs were not included since they were commonly used for several other indications and could be obtained without a prescription.

Increase in therapy was defined as any increase in the medications of interest regardless of any concomitant reduction in other medications. Decrease in therapy was defined as any decrease in the medications of interest without any concomitant increase in other medications. However, change in therapy was not just a simple change in the dosage of the medications. Several special circumstances had to be taken into account. For some immunosuppressive agents, different dosing levels based on body weight were used in the definition of change in therapy. A change in therapy was deemed to have occurred when there was a change in the dosing level of these medications. A change in immunosuppressive therapy was generally considered to be an increase in therapy, except in the case of changing from cyclophosphamide to azathioprine, methotrexate, or cyclosporin A. This is because it is common practice to make such a change once the disease is under control (the step-down phase), since prolonged cyclophosphamide therapy is associated with significant toxicity. Therefore, this step-down phase was equivalent to a reduction in therapy, since the discontinuation of cyclophosphamide was considered to be a decrease in therapy while the initiation of the other immunosuppressive agent was not considered an increase.

When treatment was changed from cyclophosphamide to mycophenolate mofetil, the local investigator was contacted to determine whether the change was a result of treatment failure with cyclophosphamide (indicating increase in therapy) or was used in a step-down phase (indicating no increase in therapy). If an immunosuppressive agent was started for steroid-sparing effect, this was not considered to be an increase in therapy. Anticoagulation therapy had to be initiated due to active disease (which was clarified with the local investigator) and in the presence of immunosuppressive agents or high-dose steroids, in order to be considered an increase in therapy.

Because most immunosuppressive agents have potential toxic effects, it is common practice to start treatment at a low dosage and gradually escalate to the target dosage. To take this into account, any increase in the dosage of immunosuppressive agents within the first 3 months of initiation was considered to be part of an escalation plan to achieve the target dose and was not considered an increase in therapy. Similarly, it is common practice to gradually reduce the glucocorticoid dosage during this period as part of the escalation plan. Therefore, any concomitant reduction in glucocorticoid dosage during the escalation phase was not considered to be a reduction in therapy. The reduction or discontinuation of any treatment due to side effects was not considered to be a reduction in therapy.

For this analysis, disease activity was divided into active disease (scores of A or B on the BILAG-2004 index or the Classic BILAG index) and minimal activity (scores of C or D on the BILAG-2004 index or the Classic BILAG index). Similarly, change in therapy was classified into 2 categories, “increase in therapy” and “no increase in therapy.” Therefore, “no increase in therapy” represented a combination of no change and decrease in therapy. The categories were defined in this way because increase in therapy was a better marker of disease activity than decrease in therapy. In practice, increase in therapy is very likely to occur with active disease and is unlikely to occur with inactive disease. The reverse cannot be said for reduction in therapy, since this is less likely to occur with inactive disease when the patient is receiving minimal therapy (such as low-dose glucocorticoids).

Maximum-likelihood logistic regression with robust variance estimation (Huber/White/sandwich variance estimator) was used with increase in therapy as the outcome variable and overall BILAG-2004 score as the explanatory variable. Results were reported as ORs and 95% CIs. Sensitivity, specificity, positive predictive value (PPV), and negative predictive value (NPV) of the overall BILAG-2004 score against the criterion were calculated from the regression analysis. For calculation of sensitivity, only observations which recorded an increase in treatment were used. A logistic regression model (with robust variance estimation) was fitted to the subset of data with overall BILAG-2004 scores as the response variable, and the intercept term was determined. This intercept term represents the sensitivity, and the 95% CI was calculated using the robust estimate of the intercept's standard error. Specificity, PPV, and NPV were calculated in a similar manner, using only a subset of observations (those indicating no increase in treatment, those indicating active disease, or those indicating minimally active disease, respectively). A similar analysis was performed using the Classic BILAG index for comparison.

## RESULTS

### Patients

A total of 1,510 assessments were obtained in 369 SLE patients. The mean ± SD age of the patients was 41.6 ± 13.2 years, and the mean disease duration was 8.8 ± 7.7 years. Most of the patients (92.7%) were women. The majority of the patients (59.9%) were white, 18.4% were Afro-Caribbean, and 18.4% were South Asian. More than 1 assessment was obtained from 88.6% of the patients during the study period. The distribution of disease activity and constructs (cross-tabulated against disease activity) are summarized in Tables 1 and 2.

**Table 1:** Distribution of disease activity scores on the BILAG-2004 index, Classic BILAG index, and SLEDAI-2K*

Disease activity score	No. (%) of assessments (n = 1,510 assessments in 369 patients)
Overall score on the BILAG-2004 index	
A	98 (6.5)
B	390 (25.8)
C	721 (47.8)
D	301 (19.9)
Overall score on the Classic BILAG index	
A	95 (6.3)
B	435 (28.8)
C	834 (55.2)
D	146 (9.7)
SLEDAI-2K score	
>12	28 (1.9)
4–12	539 (35.7)
1–3	419 (27.7)
0	524 (34.7)

* The overall score on the British Isles Lupus Assessment Group 2004 (BILAG-2004) index and on the Classic BILAG index was the highest score achieved in any system in the index. SLEDAI-2K = Systemic Lupus Erythematosus Disease Activity Index 2000.

**Table 2:** Cross-tabulation of overall scores on the BILAG-2004 index with constructs (ESR, anti-dsDNA level, C3 level, C4 level, and SLEDAI-2K score)*

	Overall score on the BILAG-2004 index			
	
Construct	A	B	C	D
ESR				
Normal (0–30 mm/hour)	29	158	267	100
Elevated (31–60 mm/hour)	23	60	70	15
Markedly elevated (>60 mm/hour)	13	25	20	7
Anti-dsDNA level				
Normal	49	228	506	216
Elevated	25	100	119	66
Very high (<5 times the ULN)	15	38	42	9
C3 level				
Normal	61	279	579	270
Very low (less than or equal to half the LLN)	4	4	6	0
Low	29	100	106	25
C4 level				
Normal	44	218	467	243
Very low (less than or equal to half the LLN)	25	49	51	6
Low	18	76	132	37
SLEDAI-2K score				
Inactive (0)	2	22	306	194
Mildly active (1–3)	4	93	240	82
Active (4–12)	75	264	175	25
Very active (>12)	17	11	0	0

* Values are the number of assessments. Data were available on erythrocyte sedimentation rates (ESRs) for 787 assessments, anti–double-stranded DNA (anti- dsDNA) levels for 1,413 assessments, C3 levels for 1,463 assessments, C4 levels for 1,366 assessments, and Systemic Lupus Erythematosus Disease Activity Index 2000 (SLEDAI-2K) scores for all 1,510 assessments. BILAG-2004 = British Isles Lupus Assessment Group 2004; ULN = upper limit of normal; LLN = lower limit of normal.

### Constructs

#### ESR

ESRs were available for 787 assessments (52.1%). There was a significant association between increasing ESR and overall BILAG-2004 scores reflecting higher disease activity (Table 3). The 2 degrees of freedom test for an association between overall BILAG-2004 score and ESR was statistically significant (*P* < 0.001).When ESR was analyzed as a continuous variable, the result was similar (*P* = 0.0002).

**Table 3:** Association of ESR, anti-dsDNA level, C3 level, C4 level, and SLEDAI-2K score with higher overall scores on the BILAG-2004 index*

Construct	OR (95% CI)
ESR	
Elevated (31–60 mm/hour)	2.1 (1.4–3.0)
Markedly elevated (>60 mm/hour)	2.9 (1.4–6.2)
Anti-dsDNA level	
Elevated	1.5 (0.99–2.1)
Very high (>5 times the ULN)	2.7 (1.6–4.8)
C3 level	
Very low (less than or equal to half the LLN)	5.0 (1.6–15.5)
Low	2.5 (1.8–3.5)
C4 level	
Very low (less than or equal to half the LLN)	4.2 (2.5–6.9)
Low	1.7 (1.2–2.3)
SLEDAI-2K score	
Mildly active (1–3)	3.0 (2.1–4.4)
Active (4–12)	20.0 (13.6–29.5)
Very active (>12)	232.7 (108.4–499.2)

* The overall score on the BILAG-2004 index was the highest score achieved in any system in the index. OR = odds ratio; 95% CI = 95% confidence interval (see Table 2 for other definitions).

#### Anti-dsDNA level

Anti-dsDNA levels were available for 1,413 assessments (93.6%). Increasing levels of anti-dsDNA were significantly associated with overall BILAG-2004 scores reflecting high disease activity (Table 3). The 2 degrees of freedom test for an association between overall BILAG-2004 score and anti-dsDNA was statistically significant (*P* = 0.0008).

#### C3 and C4 levels

C3 and C4 levels were available for 1,463 assessments (96.9%) and 1,366 assessments (90.5%), respectively. There was a significant association between lower C3 levels and overall BILAG-2004 scores reflecting higher disease activity and between lower C4 levels and overall BILAG-2004 scores reflecting higher disease activity (Table 3). For both models, the 2 degrees of freedom test was statistically significant (*P* < 0.0001).

#### SLEDAI-2K score

SLEDAI-2K scores were available for all assessments. Higher SLEDAI-2K scores were significantly associated with overall BILAG-2004 scores reflecting higher disease activity (Table 3). The 3 degrees of freedom test for an association between overall BILAG-2004 score and SLEDAI-2K score was significant (*P* < 0.001). Results were similar when SLEDAI-2K score was analyzed as a continuous variable (*P* < 0.0001).

#### Multivariate analysis

For completeness, we performed a multivariate analysis with ESR, anti-dsDNA level, C3 level, and C4 level included in the same regression model. Only increasing ESR and low C4 level remained significantly associated with overall BILAG-2004 scores reflecting higher disease activity.

### Criterion validity

Of the 1,510 assessments, 342 (22.6%) resulted in an increase in therapy, 320 (21.2%) resulted in a decrease in therapy, and 848 (56.2%) were not followed by a change in therapy (Table 4). The odds of an increase in therapy were higher with overall BILAG-2004 scores reflecting higher disease activity (Table 5).

**Table 4:** Cross-tabulation of overall scores on the BILAG-2004 index with change in therapy*

	Change in therapy		
	
Overall score on the BILAG-2004 index	Decrease	No change	Increase
D	83	213	4
C	197	464	61
B	38	147	205
A	2	24	72

* Values are the number of assessments. BILAG-2004 = British Isles Lupus Assessment Group 2004.

**Table 5:** Association of increase in therapy with overall score on the BILAG-2004 index*

Overall score on the BILAG-2004 index	OR (95% CI)
A	204.9 (65.6–639.9)
B	82.0 (29.9–225.0)
C	6.8 (2.4–19.1)

* BILAG-2004 = British Isles Lupus Assessment Group 2004; OR = odds ratio; 95% CI = 95% confidence interval.

### Sensitivity and specificity

Scores indicating active disease (overall scores on the BILAG-2004 of A and B) were significantly associated with an increase in therapy (OR 19.3 [95% CI 14.1–26.4]). The sensitivity, specificity, PPV, and NPV of the BILAG-2004 index are summarized in Table 6. The BILAG-2004 index and the Classic BILAG index had equivalent sensitivity, specificity, PPV, and NPV.

**Table 6:** Sensitivity, specificity, positive predictive value, and negative predictive value of the BILAG-2004 index and Classic BILAG index*

	BILAG-2004 index	Classic BILAG index
Sensitivity, %	81.0 (76.4–84.9)	83.6 (79.1–87.3)
Specificity, %	81.9 (78.4–85.0)	79.1 (75.5–82.3)
PPV, %	56.8 (51.7–61.7)	54.0 (49.3–58.6)
NPV, %	93.6 (91.9–95.0)	94.3 (92.6–95.6)

* Values in parentheses are the 95% confidence interval. BILAG = British Isles Lupus Assessment Group; PPV = positive predictive value; NPV = negative predictive value.

## DISCUSSION

The results of this large multicenter cross-sectional study demonstrated the validity of the BILAG-2004 index as a measure of SLE disease activity, based on its construct and criterion validity. Construct validity was confirmed by the expected association between index scores and the ESR, C3 level, C4 level, anti-dsDNA level, and SLEDAI-2K score. Criterion validity was confirmed by the increasing strength of association between BILAG-2004 scores reflecting increasing disease activity and increase in therapy.

The results of the multivariate analysis of construct validity were rather surprising, since we expected elevated anti-dsDNA level and/or C3 level, instead of elevated ESR and C4 level, to remain significantly associated with increasing overall scores on the BILAG-2004 index. Because this was a cross-sectional study, it was not possible to determine why there was an association between increased disease activity in SLE, as measured by the BILAG-2004 index score, and low C4 level but not low C3 level in the multivariate analysis. It should be noted that low levels of C4 have previously been found to be a predictor of renal flare (9). Furthermore, low C4 levels have been found to be associated with the presence of anti-Ro antibodies and major histocompatibility complex haplotype B8;C4AQ0;DR2;DQ2, which could predispose to skin, pulmonary, and neurologic involvement (10–15). A longitudinal study is needed to determine whether there is an association between a reduction in C4 levels and an increase in disease activity in SLE as measured by the BILAG-2004 index.

The BILAG-2004 index was developed as an ordinal scale index, and the scores for individual systems were not intended to be summed into a global score. Therefore, for the purpose of validation, the best way to represent overall disease activity in any individual patient was to use the highest score achieved in any system within the index. This is logical, since a patient with any system scoring grade A or B should be categorized as having active disease (requiring therapy in principle), regardless of how many systems have a score of A or B. From an analysis viewpoint, this may put the BILAG-2004 index at a disadvantage, since there is a ceiling effect, which may underestimate the severity of the illness. For example, a patient with 5 systems scoring B will have the same overall score as a patient with only 1 system scoring B. However, for clinical trials and outcome studies, it may be appropriate to consider the number of systems with a given categorical score in the analysis.

Change in therapy was chosen as the gold standard for designating disease as active, in the absence of a better alternative. Physician's global assessment has been used previously as a benchmark, but several studies have shown this to be unsatisfactory, with poor agreement between physicians (16–19). Although the BILAG-2004 index was developed on the basis of the principle of physician's intention to treat, use of change in therapy as the criterion for active disease should not explicitly bias the analysis in favor of the index, since actual change in therapy does not determine the scoring. Only the presence of manifestations of active disease influences the scoring. Furthermore, the patient's score on the index was not available to the physician when the treatment decision was made, and it is difficult to calculate the score on the BILAG-2004 index in routine clinical practice without the appropriate reference documents.

One of the limitations of this study is inherent in the cross-sectional design, in that it only accounts for disease activity in SLE at the time of assessment. This does not take into account the level of disease activity prior to the assessment, which influences treatment decisions. The treatment decision regarding a patient with active disease is very different if prior disease activity was low (such as a change from grade D to B) when treatment would be increased, than if prior disease activity was high (such as a change from grade A to B) when treatment would not be increased and, in fact, might be decreased.

Making a treatment decision is a complex process that involves consideration of several factors apart from the physician's intent to treat. Such factors include current therapy, previous therapy (and its effect), the patient's opinion (in particular, refusal to change therapy as advised), and the presence of comorbid conditions. Unfortunately, it was not possible to model all of these factors in the present study. This may explain the relatively low PPV of the BILAG-2004 index. On the other hand, the high NPV is reassuring, since this indicates that increase in therapy is very unlikely in the absence of high disease activity as measured by the BILAG-2004 index.

It is not surprising that the BILAG-2004 index and the Classic BILAG index had similar sensitivity, specificity, PPV, and NPV, since the main difference between the 2 indices is the addition of ophthalmic and gastrointestinal manifestations in the BILAG-2004 index. Active disease manifestations in these 2 systems were not common in this study; there were only 6 assessments (from 5 patients) with a score of A or B in the gastrointestinal system and 8 assessments (from 3 patients) with a score of A or B in the ophthalmic system. Although these manifestations are uncommon, they are significant and important for individual patients and need to be captured.

In conclusion, the BILAG-2004 index is a valid measure of disease activity in SLE. It is more comprehensive, incorporates more up-to-date terminology, and has a clearer glossary of definitions than the Classic BILAG index. Therefore, we recommend that the BILAG-2004 index be considered for use in clinical trials and outcome studies of SLE.

## AUTHOR CONTRIBUTIONS

Dr. Yee had full access to all of the data in the study and takes responsibility for the integrity of the data and the accuracy of the data analysis.

**Study design.** Yee, Farewell, Isenberg, Rahman, Teh, Griffiths, Bruce, Akil, McHugh, D'Cruz, Khamashta, Maddison, Gordon.

**Acquisition of data.** Yee, Isenberg, Rahman, Teh, Griffiths, Bruce, Ahmad, Prabu, Akil, McHugh, D'Cruz, Khamashta, Gordon.

**Analysis and interpretation of data.** Yee, Farewell, Isenberg, Gordon.

**Manuscript preparation.** Yee, Farewell, Isenberg, Rahman, Teh, Griffiths, Bruce, Prabu, Akil, McHugh, D'Cruz, Khamashta, Gordon.

**Statistical analysis.** Yee, Farewell, Gordon.
